# Long-term follow-up of pilonidal sinus disease treated by radial laser surgery

**DOI:** 10.1007/s00423-024-03455-0

**Published:** 2024-08-23

**Authors:** Koskinen Karita, Lindström Adalia, Poussa Tuija, Harju Jukka, Hermunen Kethe

**Affiliations:** 1 Faculty of Medicine, University of Helsinki, Helsinki, Finland; 2 Helsinki University Hospital, Abdominal Center, Espoo, Finland; 3 STAT-Consulting, Nokia, Finland

**Keywords:** Long-term results, Long-term follow-up, Pilonidal disease, Pilonidal sinus disease, Sinus pilonidalis, Laser surgery

## Abstract

**Purpose:**

Laser ablation is one of the newest and most advanced minimally invasive techniques in treating pilonidal sinus disease (PSD). Most studies on the subject have small sample sizes and relatively short follow-up times, making evaluation of long-term healing rates and recurrences difficult. Furthermore, long-term results for laser surgery of PSD are still lacking. The aim of this study was to retrospectively report long-term follow-up results for PSD treatment by radial laser surgery.

**Methods:**

We retrospectively studied the medical records of 83 patients who underwent the radial laser procedure for PSD between January 2017 and September 2019. Our follow-up time was a median of 5.2 years, range 1.5 to 7.4 years.

**Results:**

Twelve patients had a PSD recurrence after their laser procedure, which gives a recurrence rate of 14.5% (95% CI 8.2%-23.2%). These recurrences appeared at a median 12.2 months after the laser procedure, range 4.2 to 51 months. A total of 23 patients (27.7%; 95% CI 19.0–38.0) underwent a reoperation, 11 patients due to postoperative infection or prolonged recovery and 12 patients due to PSD recurrence. Recurrent PSD and spillage of pus during operation were statistically significantly associated with the need for a second operation.

**Conclusion:**

Radial laser surgery provides a minimally invasive treatment option with an acceptable recurrence rate in long-term follow-up.

## Introduction

Pilonidal sinus disease (PSD) is a heterogeneous disease group with no consensus regarding its best treatment modality [1]. It is a fairly common disease in which treatment failure and disease recurrences are frequent [2], leading to considerable morbidity in otherwise healthy patients [3]. One meta-analysis analysing 740 studies and more than 80.000 patients on PSD surgery showed that recurrence in PSD is highly dependent on surgical procedure and by follow-up time [4]. Recurrence of PSD may occur up to 20 years after surgery [5] and follow-up of at least five years after PSD surgery is considered the gold standard [6, 7].

In recent years new minimally invasive techniques have become more popular for treating PSD [8], including pit-picking [9], treatment with phenol [10], with fibrin-glue [11], with endoscopy [12], as well as the Gips technique using trephines [13], and laser ablation [14]. Minimally invasive techniques are generally considered to be associated with reduced postoperative morbidity and quicker return to normal daily activities than more conventional surgical methods [15].

Laser ablation is one of the newest and most advanced minimally invasive techniques. In a systematic review covering nine studies of laser treatment, Romic et al. showed that 94.4% of patients could achieve primary healing with a weighted mean complication rate of 10%; with a median follow-up of 12 months, their weighted mean recurrence rate was 3.8% [16]. These results of high healing rate, low recurrence rate, and minor complications were achieved with quite small sample sizes (ranging from 20 to 237) and relatively short follow-ups (median 12 months).

Several short-term studies have compared laser treatment to other PSD surgery techniques as shown in Table 1. We could only find one prospective randomized study involving laser treatment, one combining laser treatment with pit excision (in 30 patients) and comparing it to the Karydakis technique (in 28) [17]. Recurrence rates were 3.6% after the Karydakis technique and 3.3% after pit excision and laser treatment, with a mean follow-up of 25 months (*p* = 0.737). Most studies’ small sample sizes and relatively short follow-up times make long-term healing rates and recurrences difficult to evaluate. It is also impossible to compare the results based on different studies having different follow-up times, especially when appropriate statistical methods, like survival analyses, are ignored.

**Table 1:** Previous studies comparing laser treatment to other surgery techniques

Study	Study type	Other surgical technique	Sample size (laser + other technique)	Follow-up period (mean/median)	Recurrence rate (laser vs. other technique)
Yardimci et al. (2020) [17]	Prospective randomized	Karydakis technique	30 + 28	25 months	3.3% vs. 3.6%
Abdelnaby et al. (2021) [33]	Retrospective	Lay-open technique	62 + 77	12 months	9.3% vs. 0%
Algazar et al. (2022) [34]	Retrospective	Limberg flap	24 + 47	13.87 months	8.3% vs. 4.3%
Ersavas et al. (2023) [35]	Retrospective	Endoscopic treatment	37 + 36	13.5 months	8.1% vs. 11.1%

In addition to laser surgery with tract ablation, a laser can serve for hair depilation in the sacrococcygeal area before or after surgery. Studies related to the effect of laser depilation on PSD recurrence rate are heterogeneous, and according to the authors of systematic reviews hard to compare [18, 19]. Halleran et al., examining 35 studies on laser hair depilation, stated that PSD recurrence afterwards ranged from 0 to 28% during a follow-up from 6 months to 5 years [18]. Pronk et al. studied 14 studies on hair removal after PSD surgery and found a recurrence rate of 9.3% after laser hair removal versus 23.4% after traditional hair removal methods (mean follow-up, 37 months) [19].

Long-term results regarding laser surgery for PSD are still lacking. Our aim was to retrospectively report such long-term follow-up results after radial laser surgery.

## Materials and methods

This retrospective study was conducted in accordance with the Declaration of Helsinki, authorization number HUS/74/2023, 17 May 2023.

### Patients

Patients who underwent the radial laser procedure for PSD between January 2017 and September 2019 in the Day Surgery Unit of the Abdominal Centre, Helsinki University Hospital, Espoo, Finland, were eligible for this study. All patients for whom the operating surgeon considered laser surgery suitable were included in this retrospective study. Exclusion criteria were not defined or used for this observational, non-experimental study, using real-world data. Collection of patient data required examination of each patient’s medical records. Some necessary data came from our earlier study reporting short-term follow-up results after laser surgery for PSD [14]. The additional data were collected in June 2024. All patients have been followed up through the Finnish healthcare system’s electronic medical records, which provide consistent patient record data.

These data included patient demographics: age, gender, body mass index (BMI), alcohol consumption, cigarette smoking, and American Society of Anesthesiologists (ASA) score, as well as clinical characteristics, including type of anaesthesia, number of sinus openings, spillage of pus from the sinus tract during surgery, follow-up time after the procedure (short-term follow-up), healing rate at first follow-up visit 2 months postoperatively, postoperative complications, postoperative interstitial fluid spillage, length of sick leave, and recurrence-free time.

We collected the following additional data for our present PSD study with long-term follow-up results: recovery after surgery, recurrence of PSD, date of recurrence, need for a reoperation, number of visits to the out-patient clinic postoperatively, laser hair epilation, whether an abscess preceded the laser procedure, postoperative infections, diabetes, whether the laser treatment was for primary or recurrent PSD, and updated follow-up time. We define PSD recurrence as the reappearance of PSD symptoms following initial complete healing from PSD surgery, necessitating a reoperation. PSD with prolonged recovery refers to a condition where the disease does not initially heal after surgery but heals with a delay either with or without a reoperation.

### Surgical technique

The technique for the radial laser procedure has been described in detail [14]. The procedures were performed in the prone position, with a preoperative intravenous administration of antibiotics (1.5 g cefuroxime and 0.5 g metronidazole). After identification of the pit openings, they were enlarged with a clamp, and the sinus tracts were examined with a metal probe and then cleaned of debris with a clamp or a curette. Then the radial laser probe (FiLaC) was inserted into the sinus tract. An energy of 10 or 13 W was applied while the probe was being withdrawn at a rate of 1 mm per second. If the tract did not close after the first withdrawal, the procedure was repeated.

### Anaesthesia

The procedures were performed under local anaesthesia, total intravenous anaesthesia, or spinal anaesthesia. The decision as to anesthetic method depended on the surgeon, the patient, and the anaesthesiologist. Oral ibuprofen and paracetamol served for postoperative pain.

### Statistical analysis

The patient and surgery characteristics are expressed as frequency (%) for categorical and dichotomous variables, and as median (range) for continuous variables.

The primary study endpoint was need of a reoperation during the 5-year follow-up. Risk ratios (RR) and 95% confidence intervals (95% CI) were calculated to find the potential risk factors for the need of a reoperation. Fisher’s exact test was used to assess the statistical significance. Odds ratios given by logistic regression analyses were overestimated and not appropriate due to the high reoperation incidence.

Kaplan–Meier survival analysis served for estimating the recurrence-free survival and reoperation-free survival (%) at 6, 12, 24 and 60 months after surgery and groups were compared using the log-rank test. Reoperation-free survival was defined as the period from date of laser surgery to date of reoperation. Patients without a reoperation were censored at the cut-off date (June, 2024) and two patients who were lost to follow-up were censored at their last follow-up date. No deaths occurred before the cut-off date.

Recurrence of PSD and any problem with recovery (postoperative infection, prolonged recovery or a recurrence) during the 5-year follow-up were the secondary endpoints. The 5-year incidences with 95% CI are given for them.

The follow-up time was calculated from date of surgery to cut-off date or the last follow-up date.

Analyses utilized IBM SPSS Statistics for Windows (version 28.0, Armonk, NY, USA, IBM Corp.). A two-sided p-value < 0.05 was considered statistically significant.

## Results

In our retrospective long-term follow-up study, we analysed the data of 83 patients who had undergone a laser procedure for PSD. Table 2, describing patient- and surgery characteristics, shows a median age of 27 years and a male predominance of 78.3%. The patients were quite healthy (97.6% ASA 1 or ASA 2), but the proportion of smokers was high (31.3%). Of the 83 patients, 70 (84.3%) were treated for primary PSD and 13 (15.7%) for recurrent PSD. The 13 patients with a recurrent PSD had had the following previous operations: excision with direct wound closure (5 patients), pit-picking procedure (2 patients), excision with open-wound and negative pressure therapy (2 patients), excision with open-wound healing (1 patient) and Karydakis flap procedure (1 patient). One patient had had two previous excisions and one patient's previous procedure was unknown. The median time from the previous operation to the laser procedure was 3.4 years, range 3.1 months – 14.1 years.

**Table 2:** Patient and surgery characteristics in patients with pilonidal disease treated by radial laser surgery (N = 83)

*Patient characteristics*		n (%)
Age at operation, years	Median (range)	27 (18–67)
BMI, kg/m^2^	Median (range)	26.6 (17.9–42.2)
Gender	Male	65 (78.3)
	Female	18 (21.7)
ASA	1	53 (63.9)
	2	28 (33.7)
	3	2 (2.4)
Alcohol	Non-drinkers	39 (47.0)
	Drinkers	44 (53.0)
Alcohol consumption, doses*/week	Median (range)	2 (1—25)
Smoking	Non-smokers	57 (68.7)
	Smokers	26 (31.3)
Cigarettes /day	Median (range)	10 (2—25)
Diabetes	Non-diabetic	79 (95.2)
	Diabetic	4 (4.8)
Acute abscess before laser treatment	No	23 (27.7)
	Yes	60 (72.3)
*Surgery characteristics*		
Pre and/or postop hair depilation	No	61 (73.5)
	Yes	22 (26.5)
Type of PSD	Primary	70 (84.3)
	Recurrent	13 (15.7)
Spillage of pus during operation	No	68 (81.9)
	Yes	15 (18.1)
Number of sinus openings	1	15 (18.1)
	2	46 (55.4)
	3–5	22 (26.5)

**Fig. 1:**
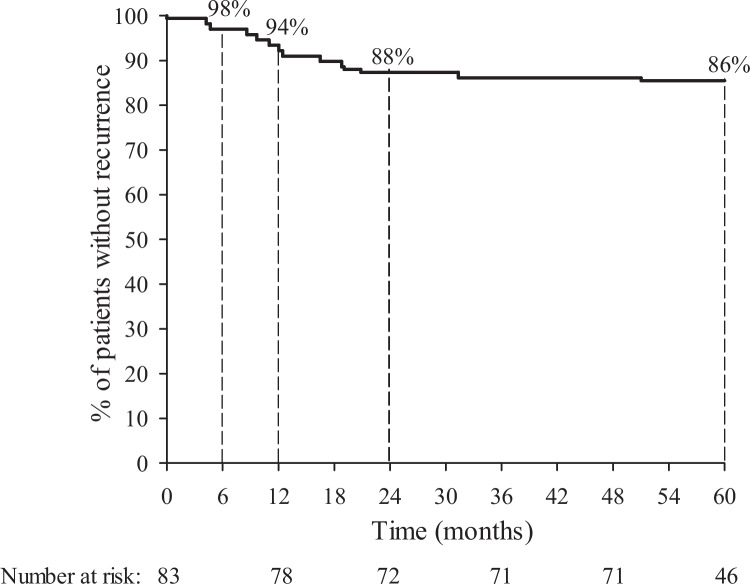
Kaplan–Meier curve showing the recurrence-free time in 83 patients with PSD treated by radial laser surgery. Percentages indicate the percentages of patients without recurrence of PSD at 6 months, and at 1, 2 and 5 years after surgery

Our follow-up (from date of laser surgery to the cut-off date) was a median of 5.2 years, range 1.5–7.4 years. The patients were divided into four groups depending on their recovery after laser surgery: 52 patients (67.2%) had complete healing, eight patients (9.6%) had a postoperative infection or a prolonged recovery without the need of a reoperation, 11 patients (13.3%) had a postoperative infection or a prolonged recovery and needed a reoperation and 12 patients (14.5%; 95% CI 8.2%-23.2%) had a recurrence of PSD and needed a reoperation. 12 recurrences appeared at a median of 12.2 months after the laser procedure, the first recurrence at 4.2 months, and all recurrences within 51 months (Fig. 1).

A total of 23 patients (27.7%; 95% CI 19.0–38.0) underwent a reoperation. These 23 operations were performed a median of 10.4 months after the first operation, range 1.7 -51.0 months. Eleven patients underwent a reoperation due to postoperative infection or prolonged recovery and 12 patients due to PSD recurrence. For patients with postoperative infection or prolonged recovery, reoperation was performed a median of 5.7 months after the first operation, range 1.7 – 9.1 months. Patients with a PSD recurrence had a reoperation on average 17.4 months (median) after surgery, range 10.4 – 51.0 months. Of these 23 patients requiring a second operation, 7 (30.4%) had a repeated laser procedure, 8 (34.8%) an open-wound and negative pressure therapy, 3 (13.0%) had a direct wound closure and 2 (8.7%) had a VY-plasty. A Karydakis flap procedure,an open-wound healing by secondary healing and an incision with drainage were each performed on one patient (4.3%). Six patients had to undergo a third operation, and two patients four operations before the PSD healed. A total of 31 patients experienced either PSD recurrence or recovery challenges (postoperative infection or prolonged healing)) during the follow-up. The 5-year incidence was then 37.3% (95% CI 27.5–48.0%).

**Fig. 2:**
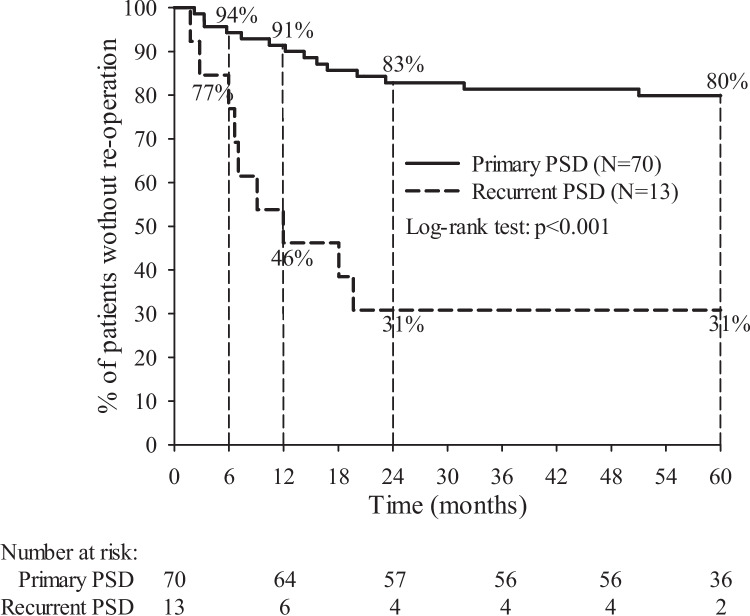
Kaplan–Meier curve showing the reoperation-free time in 83 patients with PSD treated by radial laser surgery according to type of PSD. Percentages indicate the percentages of patients without re-operation at 6 months, and at 1, 2 and 5 years after surgery

Table 3 shows associations between patient and surgery characteristics and the need of a reoperation. The type of PSD (primary vs. recurrent) and spillage of pus during operation were statistically significantly associated with the need for a second operation. In all, 20.0% of the patients treated for primary PSD needed a reoperation vs. 69.2% of the patients treated for a recurrent PSD (*p* < 0.001). Figure 2 shows the well-separated Kaplan–Meier curves for reoperation-free survival (%) during the five years of follow-up between primary PSD vs. recurrent PSD. Also, 22.1% of patients without spillage of pus during the operation needed a reoperation vs. 53.3% of patients with spillage of pus during the operation (*p* = 0.02).

**Table 3:** Risk ratios (RR) to assess associations between patient and surgery characteristics and need of reoperation

			Re-operation		Risk ratio		
*Patient characteristics*		N	N%	%	RR	95% CI	*p*-value
Age, years	≤ 26	43	12	27.9	Ref.^*^		
	≥ 27	40	11	27.5	0.99	0.49—1.98	1.00
BMI, kg/m^2^	< 25.0	29	7	24.1	Ref		
	25.0–29.9	43	14	32.6	1.35	0.62—2.93	0.60
	≥ 30.0	11	2	18.2	0.75	0.18—3.09	1.00
Gender	male	65	17	26.2	Ref		
	female	18	6	33.3	1.27	0.59—2.75	0.56
ASA	1	53	14	26.4	Ref		
	2–3	30	9	30.0	1.14	0.56—2.30	0.80
Alcohol	Non-drinker	39	13	33.3	Ref		
	Drinker	44	10	22.7	0.68	0.34—1.38	0.30
Smoking	Non-smoker	57	15	26.3	Ref		
	Smoker	26	8	30.8	1.17	0.57—2.41	0.79
Diabetes	Non-diabetic	79	21	26.6	Ref		
	Diabetic	4	2	50.0	1.88	0.66—5.36	0.31
Acute abscess before	No	24	7	29.2	Ref		
laser treatment	Yes	59	16	27.1	0.93	0.44—1.97	1.00
*Surgery characteristics*							
Pre and/or postop hair	No	61	14	23.0	Ref		
depilation	Yes	22	9	40.9	1.78	0.90—3.52	0.16
Type of PSD	Primary	70	14	20.0	Ref		
	Recurrent	13	9	69.2	3.46	1.91—6.26	< 0.001
Spillage of pus	No	68	15	22.1	Ref		
during operation	Yes	15	8	53.3	2.42	1.26—4.64	0.02
Number of sinus openings	1	15	5	33.3	Ref		
	2	46	11	23.9	0.72	0.30—1.73	0.51
	3–5	22	7	31.8	0.95	0.37—2.45	1.00
Surgeon	Senior	71	19	26.8	Ref		
	Junior	12	4	33.3	1.25	0.51—3.03	0.73

^*^ Ref. = reference group

Of our 83 patients, 22 (26.5%) had been treated with laser hair depilation: 6 had the treatment before the procedure, 13 patients afterwards, and 3 both before and after. These patients received the treatment 2 times, (median, range 1–8). Hair depilation did not significantly associate with study endpoints.

## Discussion

Our long-term study revealed that laser surgery, after a median follow-up of 5.2 years, had a recurrence rate of 14.5%, and recurrence could occur up to 51 months after surgery. Of our patients 27.7% needed a reoperation. Recurrent PSD and spillage of pus during operation were statistically significantly related to the need for a reoperation. Patients operated on for recurrent disease had a 3.46 times higher risk of needing a reoperation compared to those operated on for primary disease.

In our earlier study, with the same patient population, we found that laser treatment is quick and safe and offers rapid patient recovery [14]. One of the benefits of laser surgery is its simplicity: it is easily repeatable in cases with recurrence if considered reasonable based on PDS severity and patient characteristics. Two recent short-term follow-up studies with relatively large sample sizes of 226 and 311 patients have noted that, to achieve a sufficiently good healing rate, laser treatment should be repeated, several times if necessary [20, 21]. Sluckin et al. reported, with the 311 patients and a median follow-up of 10 months, an initial success rate of 66% after one laser procedure and 92% and 98% after two and three procedures [20]. De Decker et al. showed, with 226 patients and a median follow-up of 129 months, a healing rate of 78.8% after one laser procedure, increasing to 85.4% after one or more procedures [21].

No long-term follow-up results are available (with which to compare ours). The tendency of PSD to recur necessitates long-term follow-up studies. With regard to mini-invasive techniques, we only found long-term follow-up studies for pit-picking and endoscopic techniques [22–26]. For these mini-invasive techniques, recurrences seem to be relatively more frequent in long-term than in short-term follow-up [27, 28].

As for our long-term results in comparison to available short-term (approximately one year) follow-up studies, Pappas and Christodoulou, with 237 patients, reported a recurrence rate of 2.9% with a median follow-up time of 354 days [29], and Dessily et al. with 200, showed a recurrence rate of 14.9% with a mean follow-up time of 525 days [30]. Our recurrence rate was 14.5%, corresponding well to that of the Dessily group. However, in our long-term follow-up, the need for a reoperation, which also takes into account those patients who needed a reoperation due to postoperative infection or prolonged recovery, is significantly higher, that is 27.7%.

Horesh et al. reported an 81.3% complete healing rate following laser surgery after a median follow-up of 24.2 months, but they provided no recurrence rate [31]. Yardimci et al., following a combination of laser treatment with pit excision after a mean follow-up time of 25 months, reported a recurrence rate of 3.3%, but this study only had 30 patients in the laser group [17]. It is noteworthy, however, that they achieved their low recurrence rate by combining laser treatment with pit excision. We only enlarged pits with a clamp, but the Yardimci group excised the external sinus orifices via a punch-biopsy scalpel with a 1-mm skin margin. It is well known that to reduce the risk of PSD recurrence, careful cleansing of the cavities is important [32]. Perhaps such tract cleansing is also more complete if the sinus orifices are totally removed. It should also be taken into account that the Yardimci group gave oral antibiotics to their patients one week before and one week after surgery whereas we did no such thing [17].

We found that the need for a reoperation was statistically significantly smaller for patients with primary PSD and without spillage of pus during operation. Based on this, the laser surgery may not be the best procedure for patients whose anatomy of the natal cleft area has already been altered by previous surgery. However, since the treatment of a recurrent disease is always challenging, the laser procedure should not be completely excluded, considering how quick and easy the procedure is. It can be a good option also in recurrent disease, especially for patients with high morbidity. These findings also indicate that no single technique is optimal for PDS, this disease being so heterogeneous. This is also supported by the previous research findings that laser treatment is most suitable for patients with a solitary cavity with a diameter of less than 2 cm [29] and without secondary openings laterally [30]. Since it seems likely that laser treatment is most suitable for small and simple cases, a classification system for PDS severity would be of considerable help for the surgeon in the preoperative assessment.

The retrospective nature of this study is its main limitation. Another limitation is the rather small number of patients.The long follow-up time is the main strength, follow-up times in other studies being much shorter. Other strengths are the Finnish healthcare system’s electronic medical records, which provide consistent patient record data.

## Conclusion

Radial laser surgery provides a minimally invasive treatment option which is quick and easy to perform and is followed by an acceptable recurrence rate, long-term. Patients recover quickly and return to normal activities within a few days.

## Data Availability

No datasets were generated or analysed during the current study.
